# Applied Sport Science for Male Age-Grade Rugby Union in England

**DOI:** 10.1186/s40798-020-0236-6

**Published:** 2020-02-21

**Authors:** Kevin Till, Jonathon Weakley, Dale B. Read, Padraic Phibbs, Josh Darrall-Jones, Greg Roe, Sarah Chantler, Stephen Mellalieu, Michael Hislop, Keith Stokes, Andrew Rock, Ben Jones

**Affiliations:** 1 Carnegie Applied Rugby Research (CARR) Centre, Institute for Sport, Physical Activity and Leisure, Leeds Beckett University, Leeds, West Yorkshire UK; 2 Leeds Rhinos RLFC, Leeds, UK; 3 Yorkshire Carnegie RUFC, Leeds, UK; 4 School of Behavioural and Health Sciences, Australian Catholic University, Brisbane, Australia; 5 Leinster Rugby, Belfield, Dublin, Republic of Ireland; 6 Bath RUFC, Bath, UK; 7 Cardiff Metropolitan University, Cardiff, UK; 8 World Rugby, Dublin, Ireland; 9 University of Bath, Bath, UK; 10 Rugby Football Union, London, UK; 11 England Performance Unit, Rugby Football League, Red Hall, Leeds, UK; 12 School of Science and Technology, University of New England, Armidale, NSW Australia; 13 Division of Exercise Science and Sports Medicine, Department of Human Biology, Faculty of Health Sciences, The University of Cape Town and the Sports Science Institute of South Africa, Cape Town, South Africa

## Abstract

Rugby union (RU) is a skill-collision team sport played at junior and senior levels worldwide. Within England, age-grade rugby governs the participation and talent development of youth players. The RU player development pathway has recently been questioned, regarding player performance and well-being, which sport science research can address. The purpose of this review was to summarise and critically appraise the literature in relation to the applied sport science of male age-grade RU players in England focussing upon (1) match-play characteristics, (2) training exposures, (3) physical qualities, (4) fatigue and recovery, (5) nutrition, (6) psychological challenges and development, and (7) injury. Current research evidence suggests that age, playing level and position influence the match-play characteristics of age-grade RU. Training exposures of players are described as ‘organised chaos’ due to the multiple environments and stakeholders involved in coordinating training schedules. Fatigue is apparent up to 72 h post match-play. Well-developed physical qualities are important for player development and injury risk reduction. The nutritional requirements are high due to the energetic costs of collisions. Concerns around the psychological characteristics have also been identified (e.g. perfectionism). Injury risk is an important consideration with prevention strategies available. This review highlights the important multi-disciplinary aspects of sport science for developing age-grade RU players for continued participation and player development. The review describes where some current practices may not be optimal, provides a framework to assist practitioners to effectively prepare age-grade players for the holistic demands of youth RU and considers areas for future research.

## Key Points

- Age, playing level and position influence the match-play characteristics and training exposure of age-grade RU players within England. Coaches and administrators should understand the complexity of match-play and training within age-grade RU and carefully plan and schedule competition and training to optimise long-term player development and participation within the sport.
- A broad range of physical qualities including body size, speed, change of direction speed, high-intensity running ability and muscular strength and power are important for player development alongside injury prevention and should be strongly considered within the programmes of age-grade RU players.
- The consideration of training exposure, fatigue and recovery, physical development, nutrition, psychological development and injury management are key topics that inform coach and key stakeholders education for maximising participation and long-term player development of age-grade RU players.

## Introduction

Rugby union (RU) is a field-based team sport with over 8.5 million players in member unions played across youth to senior and amateur to international levels worldwide [1]. The sport is a contact-skill-based, intermittent, high-intensity invasion sport, involving periods of static exertions, collisions and running, interspersed with variable periods of lower intensity work and rest [2–5]. At the senior level, RU is contested between two teams over two 40-min halves separated by a 10–15 min break, with reduced playing time for junior levels dependent upon age [6]. The ultimate aim of a match is to score a greater number of points than the opposition in accordance with the laws of the game that are enforced by World Rugby, the international governing body of RU. A RU team consists of 15 players and a maximum of eight replacements, totalling a 23-man squad. Players are commonly split into two positional sub-groups (‘backs’ or ‘forwards’) or six sub-positions of front row (‘prop’, ‘hooker’), second row, back row (‘flanker’, ‘number eight’), scrum-half, inside backs (‘fly-half’, ‘inside centre’, ‘outside centre’) and outside backs (‘fullback’, ‘wing’). Typically, backs perform more running, whilst forwards undertake increased collision and contact activities [7].

Rugby union participation is higher in England than any other nation [8] with an estimated total of 2.1 million players [1]. England has its own structure of youth RU, known as age-grade rugby, whereby players participate within annual-age categories (e.g. under 13 years of age [U13], under 18 years of age [U18]). England RU’s national governing body, the Rugby Football Union (RFU), governs age-grade rugby in relation to participation within the game alongside the identification and development of young talented players. Talent identification and development programmes are delivered via fourteen Regional Academies, normally aligned with professional RU clubs. Players are typically identified from community or school rugby and invited to train within a Regional Academy from 15 years of age, prior to potentially signing a professional contract at 18 years of age. Players may remain in an academy programme until their early twenties. Between 15 and 18 years of age, RU players may train and compete within multiple rugby programmes (i.e. club, school, representative and [regional] academy) alongside undertaking other sporting activities and school-based commitments (e.g. Physical Education [9, 10]). Therefore, RU within England employs a late specialisation model [11], especially compared to other sports (e.g. soccer [12]), resulting in a complex multi-sport, multi-environment and multi-coach development programme. This programme has been previously termed as ‘organised chaos’, whereby ‘organised’ is defined as making arrangements or preparations for an event, and ‘chaos’ is defined as the property of a complex system whose behaviour is so unpredictable it appears random [13].

Recent consensus statements [14–16] suggest youth (or long-term) athletic development programmes should aim to develop healthy, capable and resilient young athletes whilst attaining widespread, inclusive, sustainable and enjoyable participation and success across all levels of individual athletic achievement. Combined with England Rugby’s aim to ensure all players enjoy rugby in a safe environment and develop a wide array of skills [17], this demonstrates that healthy youth athletic development is a necessity for all age-grade rugby players. Therefore, sustainable participation and player development within age-grade RU players is a focus for the RFU and World Rugby. However, due to the complex multi-sport and multi-environment playing system within RU, questions have been raised regarding player wellness and performance to maintain participation and support player development towards the elite level within the sport [10].

Therefore, the purpose of this review article was to summarise and critically appraise the scientific literature in relation to the applied sport science of male age-grade RU focussing upon England. This included (1) match-play characteristics, (2) training exposures, (3) physical qualities, (4) fatigue and recovery, (5) nutrition, (6) psychological challenges and development and (7) injury. The review focussed upon RU in England based upon the structure of their age-grade programme and the importance of context within sport science [18]. There are differences in the player development systems applied worldwide (e.g. concurrent playing pathways, age player obtains professional contract, academy structure and support, sport governance) with RU Nations (e.g. New Zealand and South Africa [19]). This review provides a framework to assist practitioners to effectively prepare age-grade players for the holistic demands, whilst considering areas for future research to enhance applied sport science within youth RU.

## Methods

To carry out this review, a computer literature search of PubMed, Google Scholar and Scopus was performed for English-language peer-reviewed articles from inception to January 2019 using the following key words and appropriate Boolean (AND/OR) phrases: ‘Rugby Union’, ‘Youth’, ‘Junior’, Adolescent’, ‘Age-Grade’, ‘Match Demands’, ‘Match Characteristics’, ‘Training’, ‘Training Load’, ‘Training Exposure’, ‘Anthropometric’, ‘Body Composition’, ‘Strength’, ‘Power’, ‘Speed’, ‘Aerobic Capacity’, ‘Fatigue’, ‘Recovery’, ‘Nutrition’, ‘Psychological Development’, ‘Psychological Challenges’ and ‘Injury’. The electronic search was supplemented by hand searching the reference lists of articles, which met the study’s inclusion criteria.

The themes of the review represented the major applied sport science themes influencing age-grade RU performance including match-play characteristics, training exposure, physical qualities, fatigue and recovery, nutrition, psychological challenges and development and injury. As the review sought to identify the applied sport science of male age-grade RU players within England, studies that investigated youth or age-grade RU players from different nations were excluded from the data tables but were discussed in the text. Studies were considered age-grade if they did not include adult rugby and therefore included studies at university and U20.

## Match-Play Characteristics

In recent years, there has been an increase in research studies evaluating the match-play characteristics of team sports [20]. Such research is intended to inform training prescription whilst understanding the match-play characteristics within youth athlete development systems. Studies within senior [21–24] and youth [25–29] RU have been conducted using video-based time motion analysis or microtechnology devices including Global Positioning Systems (GPS). Specific to male age-grade RU match-play within England, nine studies have been conducted across school [5, 9, 30], county representative [3], university [5], academy [9, 30–34] and international [35] playing levels. Table 1 summarises the locomotor-related variables whilst Table 2 shows the speed threshold and PlayerLoad-related variables for physical match-play characteristics.

**Table 1:** The locomotor characteristics of young rugby union players during match-play within England

Study	Level of play	Age	Position	Sample size (*n*)	Playing time (min)	Total distance (m)	Average speed (m min^−1^)
Cunningham et al. [35]	International	U20	Forwards	21 (81)	87.6 ± 9.7	5370 ± 830	61.5 ± 8.0
			Backs	19 (80)	90.4 ± 8.1	6230 ± 800	69.1 ± 7.6
Phibbs et al. [9]	Academy	U18	Forwards	16	62.9 ± 17.8	4128 ± 1232	65.0 ± 5.7
			Backs	15	69.2 ± 0.2	4770 ± 741	69.4 ± 5.5
	School	U18	Forwards	15	61.1 ± 16.9	3884 ± 1255	58.7 ± 8.1
			Backs	15	65.5 ± 14.0	4457 ± 1009	66.9 ± 8.4
Read et al. [5]	School	U16	Forwards	16	62.5 ± 2.3	4364 ± 654	69.7 ± 9.2
			Backs	15	58.8 ± 7.8	3841 ± 700	66.4 ± 9.4
		U18	Forwards	18	66.2 ± 15.5	4232 ± 985	64.2 ± 5.4
			Backs	16	65.7 ± 17.8	4489 ± 1299	68.3 ± 5.7
	University		Forwards	17	70.7 ± 21.4	4683 ± 1377	66.6 ± 5.0
			Backs	14	82.4 ± 10.7	5889 ± 719	71.1 ± 5.5
Read et al. [3]	County	U16	Forwards	20	49.3 ± 18.5	–	77.8 ± 5.4
			Backs	15	52.1 ± 20.3	–	79.8 ± 10.5
		U18	Forwards	21	51.1 ± 19.4	–	74.9 ± 6.8
			Backs	19	52.9 ± 18.4	–	78.7 ± 7.0
		U20	Forwards	18	59.9 ± 22.8	–	65.3 ± 3.2
			Backs	19	61.8 ± 23.2	–	70.9 ± 8.7
Read et al. [30]	Academy	U18	Forwards	7 (21)	76.4 ± 3.7	5461 ± 360	71.7 ± 6.6
			Backs	12 (24)	76.4 ± 3.8	5639 ± 368	74.0 ± 6.6
	School	U18	Forwards	25	74.1 ± 4.1	4881 ± 388	66.0 ± 5.0
			Backs	25	74.2 ± 3.8	5260 ± 441	71.0 ± 5.4
Roe et al. [33]	Academy	U18	Forwards	12 (43)	66.0 ± 13.0	4747 ± 1002	–
			Backs	14 (38)	70.0 ± 11.0	5201 ± 810	–
Roe et al. [32]	Academy	U18	All	14	73.6	4691 ± 878	74.0 ± 6.0

Data are displayed as mean ± SD. Sample size is the number of participants in the study, followed by the total number of observations in brackets if different

**Table 2:** The speed threshold and PlayerLoad characteristics of young rugby union players during match-play within England

Study	Level of play	Age	Position	Sample size (*n*)	Speed thresholds				PL	PL_slow_
					Threshold 1	Threshold 2	Threshold 3	Threshold 4		
Cunningham et al. [35]								> 5 m s^−1^		
	International	U20	Forwards	21 (81)				284 ± 135 m		
			Backs	19 (80)				656 ± 183 m		
Phibbs et al. [9]						< 61% MSS	61–90% MSS	≥ 90% MSS	AU	
	Academy	U18	Forwards	16		3901 ± 1202 m	220 ± 111 m	5 ± 10 m	420 ± 130 AU	
			Backs	15		4489 ± 720 m	280 ± 96 m	15 ± 15 m	431 ± 98 AU	
	School	U18	Forwards	15		3698 ± 1217 m	138 ± 114 m	0 ± 1 m	399 ± 141 AU	
			Backs	15		4098 ± 918 m	359 ± 182 m	19 ± 24 m	378 ± 86 AU	
Read et al. [5]					0–1.94 m s^−1^	1.95–3.33 m s^−1^	3.34–5.83 m s^−1^	> 5.84 m s^−1^	AU	AU
	School	U16	Forwards	16	2007 ± 218 m	1278 ± 291 m	993 ± 295 m	87 ± 86 m	456 ± 47 AU	231 ± 24 AU
			Backs	15	2011 ± 304 m	865 ± 325 m	843 ± 342 m	165 ± 101 m	332 ± 76 AU	152 ± 34 AU
		U18	Forwards	18	2099 ± 546 m	1044 ± 318 m	995 ± 370 m	94 ± 93 m	437 ± 96 AU	224 ± 51 AU
			Backs	16	2307 ± 647 m	854 ± 264 m	1009 ± 444 m	319 ± 176 m	395 ± 118 AU	172 ± 49 AU
	University		Forwards	17	2235 ± 699 m	1271 ± 400 m	1112 ± 442 m	64 ± 65 m	504 ± 157 AU	250 ± 76 AU
			Backs	14	2820 ± 503 m	1256 ± 219 m	1460 ± 357 m	353 ± 147 m	500 ± 80 AU	213 ± 31 AU
						0–3.33 m s^−1^	> 3.34 m s^−1^		AU·min^−-1^	AU·min^−1^
Read et al. [3]	County	U16	Forwards	20		55.2 ± 4.1*	22.6 ± 2.9*		7.3 ± 0.6**	3.1 ± 0.3**
			Backs	15		52.1 ± 5.1*	27.7 ± 7.7*		6.8 ± 1.2**	2.4 ± 0.3**
		U18	Forwards	21		54.9 ± 4.3*	20.2 ± 6.9*		7.6 ± 1.0**	3.3 ± 0.3**
			Backs	19		53.2 ± 5.4*	25.5 ± 4.6*		7.2 ± 1.1**	2.7 ± 0.4**
		U20	Forwards	18		50.7 ± 4.8*	14.5 ± 3.4*		6.9 ± 0.7**	3.4 ± 0.4**
			Backs	19		50.4 ± 6.2*	20.6 ± 3.9*		6.1 ± 1.0**	2.6 ± 0.4**

Data are displayed as mean ± SD. Sample size is the number of participants in the study, followed by the total number of observations in brackets if different

*PL* PlayerLoad, *PL*_*slow*_ PlayerLoad slow, *MSS* maximal sprint speed, *AU* arbitrary units

*m min^***−***1^

**AU min^***−***1^

### Absolute and Relative Distance Measures

The total distance covered during match-play within England for age-grade RU players ranges from 3841 ± 700 m in U16 school players [5] to 6230 ± 800 m during U20 international competition [35]. Intensity, measured via average speed, ranges from 58.7 ± 8.1 m min^−1^ in U18 schoolboy forwards [9] to 79.8 ± 10.5 m min^−1^ in U16 county backs [3].

The total distance and average speed, assessed via GPS, were greater in backs than forwards [5, 9, 32, 35], which is consistent with findings in senior RU [22]. No differences were identified in average speed between positions in U16 county players [3] whilst U16 school forwards covered more total distance than the backs [5]. These findings are consistent with research in South Africa [28] suggesting that differences in position-specific physical characteristics may become more apparent as age increases. This finding might be attributable to inferior technical ability at younger age categories [36] resulting in backs having less game involvements at younger age categories.

The total distance typically increases with age although it appears this is specific to the playing level and position. School U16 forwards covered more distance than U18 school backs [9, 30]. The greater total distances in older age categories (i.e. U20 and university) is likely because of the longer playing durations at these ages. Conversely, average speed does not seem to increase with age as during match-play U20 international players had one of the lowest average speeds [35] whilst U16 county backs had the highest [3]. Such findings might be apparent due to the difference in body mass between age categories and the subsequent collision characteristics, although this is yet to be confirmed. Two studies have compared the match-play characteristics between playing levels [9, 30] showing academy players had a greater total and average speed than schoolboy players. This highlights the need for appropriate player preparation strategies, as players may represent both levels concurrently.

The research reviewed above (and in Table 1) only considers the characteristics of the whole match, whereas the ‘peak’ locomotor characteristics are of likely more importance for enhancing training prescription and player development [37]. Recent research has attempted to better understand match-play characteristics by accounting for ball in play time and the peak 1-min periods. For example, the ball is in play for 37% of the match during U18 academy RU (63% ball out of play) with an average cycle (i.e. ball in play time prior to a break in play) time of 33 ± 24 s [34]. Attacking phases average speed ranged between 112.2–114.6 m min^−1^ and defensive phases ranged between 109.0–114.5 m min^−1^ [34]. Furthermore, the maximum average speed using a 0.1-s rolling mean for a 1-min period during U18 academy RU ranged between 154 ± 17 (front row) and 185 ± 20 (scrum-half) m min^−1^ demonstrating substantially greater values than those presented in whole match analysis [31]. These values can be used when planning, ‘live’ monitoring and retrospectively analysing training so players are prepared for the ‘worst-case scenario’ during matches as in recently completed studies in senior international players [7, 38].

### Speed Thresholds

Several studies have provided a breakdown of the distance covered using speed thresholds [3, 5, 9, 30, 33, 35]. Although comparisons are difficult due to the different thresholds utilised (see Table 2), findings demonstrate that most distance in RU match-play is covered at low speeds and backs cover greater distances at higher speeds compared to forwards. These findings are consistent with senior RU [22, 23] and occur due to greater running velocities in backs alongside their ability to undertake more free running in match-play. The distances covered at high speeds (e.g. > 5.84 m s^−1^) appear to increase with age in the educational pathway of school and university backs [5], whereas distance covered > 3.33 m s^−1^ by county players is similar between ages in the backs and decreases as age increases in the forwards [3]. Comparisons of speed thresholds across playing levels are difficult, but current data show similar high-speed distances between school and academy players [9, 30].

### Collisions

The collision activity of youth RU players is yet to be extensively researched. Roe et al. [33] is the only study to date that has quantified the number of collisions in U18 academy RU match-play showing forwards and backs completed a similar number of carries (4 ± 3 vs. 4 ± 2) and defensive rucks (2 ± 2 vs. 1 ± 1). However, forwards performed more attacking rucks (11 ± 6 vs. 4 ± 3) and tackles (9 ± 5 vs. 6 ± 3), alongside the addition of 14 ± 5 scrums [33].

As the coding of performance analysis variables can be time-consuming, researchers have used proxy measures of collision activity such as PlayerLoad (PL; a vector magnitude that sums the frequency and magnitude of accelerations in the three axial planes) and PlayerLoad slow (PL_slow_; data when the speed is < 2 m s^−1^). Associations between PL, PL_slow_ and collision number have been established (*r* = 0.79) [33]. Academy players accumulate greater measures of PL_slow_ than school players, potentially indicating greater collision activity [30]. Forwards accumulate greater PL and PL_slow_ during match-play than backs, and these measures also increase with age. However, it is unknown if this is due to greater playing durations at older ages or due to a greater frequency or magnitude of collisions. Whilst PL is used as a proxy measure of collisions, it also has a very strong (*r* = 0.94) association with total distance covered [33]. Therefore, differences in PL might be due to the greater locomotor characteristics, alongside collisions.

### Summary

Overall, the physical match-play characteristics that age-grade RU players are exposed to vary depending on playing level and age. Academy level RU appears to have greater physical match-play characteristics than school RU, thus players should be prepared for these match-play characteristics to ensure safe and optimal player development. Further research is required to understand the complexity of the physical match-play characteristics within RU. This will delineate the running and collision characteristics that concurrently contribute to the physical characteristics of match-play, alongside considering the technical and tactical elements.

## Training Exposure

In recent years, the focus on training monitoring of athletes has exponentially increased [39]. Within youth sport populations, research [14–16] has highlighted the importance of developing healthy, capable and resilient youth athletes, which promotes positive outcomes (e.g. enhanced fitness) whilst minimising negative consequences (e.g. injury). Such a focus has resulted in training exposure research within age-grade RU [26, 40–44]. Within England, the complex multi-sport and multi-environment may not be optimal to manage associated positive and negative outcomes. This has resulted in eight studies examining training exposure across school [9, 45–47], club [47] and academy [9, 13, 45, 47–50] players (Table 3).

**Table 3:** The training exposure of young English rugby union players

Study	Level of play	Age group	Sample size (*n*)	Study duration	Results
Palmer-Green et al. [45]	School	U18	250	2 seasons (2006–2007; 2007–2008)	72 h/season comprised of 58% rugby, 15% conditioning, 13% weights, 6% speed, 5% prehab, 3% other
	Academy	U18	222		190 h/season comprised of 37% rugby, 11% conditioning, 27% weights, 4% speed, 12% prehab, 9% other
Taylor et al. [49]	Academy	U18	10	6 weeks in season (178 training sessions/matches)	Mean weekly ***−*** training duration = 205 ± 96 min. The mean weekly internal loads were sRPE = 877 ± 273 AU, bTRIMP = 271 ± 97 AU, eTRIMP = 360 ± 104 AU, luTRIMP = 295 ± 92 AU, iTRIMP = 479 ± 199 AU. Mean weekly external loads were total distance = 9939 ± 2989 m, PL = 941 ± 324 AU, iHSD = 3081 ± 844 m, 15HSD = 2317 ± 752 m and 18HSD = 738 ± 210 m.
Roe et al. [50]	Academy	U21	14	11 weeks pre-season	Mean weekly sRPE = 1810 ± 310 AU
Weakley et al. [46]	School	U18	35	12 weeks in-season	Gym frequency = 1.4 ± 0.6, training load (sRPE) = 1014.0 ± 1016.0 AU, gym training time = 78.0 ± 33.2 min, non-gym training time = 120.0 ± 151.0 min, training time = 188.0 ± 144.0, lower body exercises completed 1.5 ± 0.8, lower body volume load completed 1967.0 ± 1352.0 kg, upper body exercises completed = 3.0 ± 1.7, upper body volume load completed = 3477 ± 2248, volume load complete = 5443.0 ± 3423.0 kg
Phibbs et al. [47]	School	U16	31	1 week in-season	Duration = 50.1 ± 6.6, sRPE = 123 ± 39, avg HR = 145 ± 8, total distance = 2672 ± 456, average speed = 54.9 ± 12.3, HSR = 751 ± 242 and PL = 262 ± 41. Duration = 56.8 ± 11.9, sRPE = 168 ± 55, avg HR = 134 ± 9, total distance = 2925 ± 467, average speed = 54.59 ± 10.4, HSR = 678 ± 179 and PL = 270 ± 42. Duration = 63.9 ± 9.7, sRPE = 231 ± 73, avg HR = 145 ± 11, total distance = 3619 ± 664, average speed = 56.8 ± 7.4, HSR = 955 ± 256 and PL = 354 ± 74. Duration = 70.3 ± 8.8, sRPE = 230 ± 67, avg HR = 148 ± 14, total distance = 3845 ± 577, average speed = 54.9 ± 7.5, HSR = 597 ± 246 and PL = 371 ± 75. Duration = 48.3 ± 5.1, sRPE = 211 ± 50, avg HR = 151 ± 12, total distance = 2903 ± 434, average speed = 59.9 ± 5.7, HSR = 590 ± 219 and PL = 316 ± 53. Duration = 62.0 ± 0.0, sRPE = 236 ± 42, avg HR = 151 ± 12, total distance = 4176 ± 433, average speed = 68.1 ± 7.3, HSR = 1279 ± 288,256 and PL = 424 ± 56.
		U18	39		
	Club	U16	36		
		U18	30		
	Academy	U16	18		
		U18	16		
Phibbs et al. [9]	School	U18 F	15	In-season (8 matches and 15 training sessions)	Duration = 76.7 ± 12.9, total distance = 3433 ± 300, LS = 3238 ± 327, HSR = 276 ± 71, VHSR = 21 ± 30, MSS = 7.1 ± 0.7 and PL = 345 ± 43. Duration = 76.7 ± 12.9, total distance = 3821 ± 386, LS = 3739 ± 197, HSR = 275 ± 105, VHSR = 4 ± 9, MSS = 7.2 ± 0.6 and PL = 350 ± 48 Duration = 68.1 ± 1.4, total distance = 4031 ± 755, LS = 3719 ± 649, HSR = 252 ± 120, VHSR = 5 ± 9, MSS = 7.2 ± 0.6 and PL = 345 ± 43. Duration = 68.3 ± 1.3, total distance = 4678 ± 356, LS = 4393 ± 348, HSR = 345 ± 160, VHSR = 5 ± 20, MSS = 7.9 ± 0.6 and PL = 476 ± 53.
		U18 B	15		
	Academy	U18 F	16		
		U18 B	15		
Phibbs et al. [48]	Academy	U18	20	10 weeks in-season (97 complete weeks)	Total duration = 301 ± 92 min, total sRPE = 1217 ± 364 AU; rugby duration = 214 ± 64 min, rugby sRPE = 845 ± 263 AU, gym duration = 72 ± 44 min, gym sRPE = 315 ± 180 AU, total distance = 11,629 ± 3445 m, VHSR = 20 ± 38 m, PL = 1124 ± 330 AU, PLslow = 542 ± 165 AU
Phibbs et al. [13]	Academy	U18	20	14 weeks in-season (1960 daily observations)	Total duration = 349 ± 128 min, total sRPE = 1425 ± 545 AU; rugby match duration = 50 ± 44 min, rugby match sRPE = 263 ± 255 AU, rugby training duration = 178 ± 115, rugby training sRPE = 662 ± 465, gym duration = 86 ± 61 min, gym sRPE = 339 ± 269 AU, other duration = 36 ± 62 min, other sRPE = 120 ± 195 AU

Data are displayed as mean ± SD. Sample size is the number of participants in the study

*F* forwards, *B* Backs, *h/season* hours per season, *sRPE* session rating of perceived exertion, *bTRIMP* bannister training impulse, *eTRIMP* Edwards training impulse, *luTRIMP* Lucia training impulse, *iTRIMP* individualised training impulse, *AU* arbitrary unit, *PL* PlayerLoad, *iHSD* individualised high speed distance (> velocity at OBLA), *15HSD* high speed distance (> 15 km/h), *18HSD* very high speed distance (> 18 km/h), *kg* kilogrammes, *avg HR* average heart rate, *LS* low speed running distance, *HSR* high speed running, distance, *VHSR* very high speed running distance, *MSS* maximal sprint speed

### All Training

Five studies [13, 45, 46, 48, 50] have quantified the total training exposure of age-grade RU players inclusive of rugby, gym and other training activity. Total training exposure was reported as 190 h per season in academy players compared to 72 h in school players [45]. The average total weekly session rating of perceived exertion (sRPE) during training was 1810 ± 391 AU for senior academy players during pre-season [50], 1014 ± 1016 AU for school players during in-season [46], 1217 ± 64 AU (excluding matches) and 1425 ± 545 AU (including matches) for academy players during in-season periods [13, 48]. Findings suggest increased training exposure at higher playing levels, as expected, with exposures for U18 players below those reported within senior RU [51, 52].

### Field Training

Field training exposure has been quantified by duration [45, 46], sRPE [13, 32, 47–49], locomotor (e.g. total distance [9, 13, 47–49]) and internal (e.g. heart rate [47] iTRIMP [49]) measures. Phibbs et al. [47] compared training exposures across age and playing levels, demonstrating that RU training duration and frequency increased with age. Training intensity was also greater at higher playing levels. Academy training was also more closely representative of match-play than schoolboy training due to position specificity [9] possibly due to greater coach experience and player ability [47]. For example, school RU backs completed less total and high-speed locomotor distance in training than match-play whilst forwards completed less low-speed activity and physical load in training. Furthermore, the peak speed achieved during training ranged from 86 to 89% of maximal sprint speeds, suggesting player opportunities to reach peak speeds are limited [9]. Therefore, coaches should consider whether the physical stimulus provided during training practices is optimal for long-term player development and preparing players for the respective match demands.

Weekly match and training exposure of academy rugby union players has been shown to be highly variable (CV = 37% [13]) with weekly total distance ranging from 7805 to 21,801 m (excluding match-play) [48]. This is due to the multiple training and sporting commitments (e.g. school, academy and club rugby) and potential variable fixture scheduling (CV = 96%) resulting in players potentially competing in none to three fixtures each week [13]. Furthermore, Taylor et al. [49] showed internal load (i.e. iTRIMP) had strong associations with changes in aerobic fitness over a 6-week period, and therefore internal HR measures may be important monitoring tools in the future. Coaches and administrators should aim to appropriately monitor and prescribe both training and competitions to reduce variability in training exposure whilst considering the importance of other training modes (e.g. gym training) for long-term athlete development and minimising injury.

### Gym Training

Five studies have considered the gym training of youth RU players [13, 45, 46, 48, 50]. Academy players have greater absolute and relative (27% of training exposure) resistance training time compared to school players (13% of training exposure) [45] with similar total percentage exposure represented in senior academy players (approximately 33–50% per week [50]). Reduced gym exposure has been shown in season within U18 (72 ± 44 min [51]; 86 ± 61 min, [13]) and school (78 ± 33 min [46]) players suggesting the focus on physical development decreases in season.

Weakley et al. [46] presented the most comprehensive description of gym exposure in age-grade RU, considering the frequency of gym sessions, exercises and volume loads of 35 players across four schools. Findings demonstrated school RU players undertook 1.4 ± 0.6 gym sessions per week comprising of 3.0 ± 1.7 and 1.5 ± 0.8 upper and lower body exercises, respectively. Consistent with field training, gym exposure was inconsistent and highly variable across the 12-week period, which may be sub-optimal for long-term physical development. The findings demonstrated strong relationships between the frequency of exercises completed and the volume load (kilogrammes lifted) with changes in physical performance across a 12-week period. This suggests gym exposure is important for physical development when appropriately planned and implemented alongside the potential to decrease injuries in RU players [45].

### Summary

Overall, training exposure of age-grade RU players increases with age and playing level but represents a highly variable structure over weekly periods previously described as ‘organised chaos’. Coaches and administrators need to consider increasing training session intensity and the inclusion of activities to elicit maximal velocities. Furthermore, the weekly and monthly training schedules of players should be designed to reduce week-to-week variability, considering the fixture schedule alongside implementation of gym exposure for the long-term development of physical qualities important for RU alongside minimising injury risk within players. Future research should continue to explore training loads of age-grade RU players whilst considering the integration of fatigue, recovery, physical development and injury within such studies.

## Physical Qualities

Due to the physical demands of RU, players require highly developed physical qualities, including anthropometry, body composition, linear and change of direction speed, high-intensity running ability, strength and power [6]. Previous research has presented the physical qualities of senior [53–56] and youth [53, 57–59] RU players across multiple ages, standards and positions. Specific to male age-grade RU players within England, ten studies [46, 49, 60–67] have presented data across various physical qualities making comparisons between age, position and playing level. Tables 4 and 5 present the physical qualities for age-grade RU players from England and provide objective markers of physical development to support talent identification and development [68].

**Table 4:** Sum of skinfolds, linear speed, momentum, change of direction speed and aerobic capacity qualities of youth rugby union players categorised by age and playing position (data presented as mean and standard deviation)

Age category	Level and position	Sample size (*n*)	Sum of 8 skinfolds (mm)	5 m (s)	10 m (s)	20 m (s)	40 m (s)	Initial sprint momentum (kg s^−1^)	505 (s)	Yo-Yo IRTL1 (m)	30:15 (km h^−1^)
Under 14	Academy [64]	5						446 ± 114			
Under 15	Academy [64]	19						529 ± 60			
Under 16	Academy [62]	29	88.8 ± 41.9	1.05 ± 0.09	1.82 ± 0.12	3.10 ± 0.19	5.66 ± 0.37	426 ± 67	*L* = 2.51 ± 0.17 *R* = 2.54 ± 0.14	1145 ± 337	18.4 ± 1.3
	Academy forwards [63]	15	109.7 ± 44.6	1.09 ± 0.11	1.88 ± 0.12	3.21 ± 0.18	5.87 ± 0.30			971 ± 328	18.0 ± 1.4
	Academy backs [63]	14	64.2 ± 20.2	1.01 ± 0.05	1.77 ± 0.08	2.99 ± 0.15	5.45 ± 0.31			1347 ± 221	18.8 ± 1.1
	Academy [64]	23						563 ± 75			
	Academy [60]	48									18.9 ± 1.1
Under 17	Academy [64]	4						609 ± 57			
Under 18	Academy [62]	23	86.7 ± 21.3	1.06 ± 0.04	1.81 ± 0.06	3.09 ± 0.12	5.51 ± 0.24	482 ± 54	*L* = 2.57 ± 0.12 *R* = 2.52 ± 0.13	1225 ± 374	18.6 ± 1.1
	Academy Forwards [63]	12	98.2 ± 20.1	1.07 ± 0.05	1.84 ± 0.06	3.14 ± 0.10	5.63 ± 0.21			1080 ± 240	18.2 ± 1.1
	Academy backs [63]	12	72.7 ± 12.9	1.05 ± 0.04	1.79 ± 0.06	3.02 ± 0.10	5.34 ± 0.17			1467 ± 451	19.2 ± 1.0
	School [65]	129			1.84 ± 0.11	3.15 ± 0.18	5.67 ± 0.31			1022 ± 515	
	Academy [65]	55			1.82 ± 0.08	3.10 ± 0.13	5.52 ± 0.27			1245 ± 451	
	Academy [61]	14		1.04 ± 0.04	1.80 ± 0.06	3.12 ± 0.10	5.57 ± 0.22				
	School [46]	35			1.90 ± 0.09	3.23 ± 0.13	5.80 ± 0.24	443 ± 58			
	Academy [60]	27									19.1 ± 1.1
Under 21	Academy [62]	15	105.3 ± 35.4	1.07 ± 0.07	1.79 ± 0.10	3.07 ± 0.13	5.43 ± 0.21	535 ± 70	*L* = 2.41 ± 0.10 *R* = 2.37 ± 0.15		
	Academy forwards [63]	9	119.4 ± 34.0	1.09 ± 0.07	1.82 ± 0.10	3.12 ± 0.11	5.52 ± 0.17				
	Academy backs [63]	6	84.1 ± 27.5	1.05 ± 0.07	1.76 ± 0.12	3.02 ± 0.15	5.32 ± 0.22				
	Academy [60]	15									19.2 + 1.0

Please note initial sprint momentum was calculated at 8–12 m [64] and 0–10 m [62], respectively

*505* agility 505 test, *Yo-Yo IRTL1* Yo-Yo intermittent recovery test level 1, *30–15* 30–15 intermittent fitness test, *L* left, *R* right

**Table 5:** Muscular strength and power qualities of youth rugby union players categorised by age, level and playing position (data presented as mean and standard deviation)

Age category	Participants including level, position and reference	Sample size (*n*)	PPO watt bike test (W)	CMJ height (cm)	3RM back squat (kg)	3RM front squat (kg)	3RM split squat (kg)	3RM bench press (kg)	3RM prone row (kg)	3RM chin up (kg)
Under 14	Academy [64]	5	1054 ± 263							
Under 15	Academy [64]	19	1208 ± 193							
Under 16	Academy [62]	29		33.5 ± 4.8						
	Academy [64]	24	1242 ± 166							
Under 17	Academy [64]	4	1443 ± 41							
Under 18	Academy [62]	23		39.5 ± 6.1		88.6 ± 10.8	*L* = 62.2 ± 13.1 *R* = 62.2 ± 13.1	82.6 ± 10.8	84.6 ± 10.8	101.0 ± 10.2
	School [65]	129						67.7 ± 15.5		90.3 ± 12.6
	Academy [65]	55						88.3 ± 12.7		96.3 ± 12.6
	School [46]	35		33.8 ± 5.20	77.4 ± 32.6			68.5 ± 12.8		88.0 ± 11.2
	School experienced *[67]	14				103.0 ± 17.4		92.1 ± 16.5		103.7 ± 14.7
	School in-experienced** [67]	11				87.5 ± 12.8		95.0 ± 13.0		73.2 ± 15.7
	School [66]	15			88.8 ± 18.8					
Under 21	Academy [62]	15		47.1 ± 3.6		118.2 ± 17.8	*L* = 112.8 ± 15.6 *R* = 113.9 ± 14.1	108.2 ± 14.1	96.8 ± 8.2	125.3 ± 13.2

*W* watts, *CMJ* countermovement jump, *3RM* three repetition maximum, *L* left, *R* right

*> 2 years resistance training experience

**> 6 to < 12 months resistance training experience

### Anthropometrics

Height and body mass have been shown to be important for RU [69]. Age-grade RU players’ height and body mass are greater for older players [62] and are higher in forwards than backs [63]. The height and body mass of U21 English players [62] appear similar to those reported for South African U20 players [59] and greater than those reported in U19 Portuguese forwards and backs [58]. Only one study has reported the anthropometric characteristics of players below 16 years old considering height, mass and maturity status in 14–17-year-old English players [64] and shows youth RU players were above the seventy-fifth and ninetieth reference percentiles for height and mass, respectively. These findings suggest advanced size and maturity may be advantageous for selection within RU, consistent with previous findings in Australia [70] which suggested measuring player height and mass prior to registration for potential player dispensation and grading.

### Body Composition

Body composition is important for performance as excessive body fat is detrimental to acceleration and the metabolic cost of exercise [6]. However, only two studies are available within English academy players [62, 63] presenting data via the sum of eight skinfolds. Findings show similar skinfolds across age categories [62] but higher skinfolds at U16 for forwards and U18 for backs [63]. Forwards have greater skinfolds compared to backs [63]. Findings are similar to studies [57, 71] utilising dual-energy X-ray absorptiometry presenting body fat percentage values of 13–14% and 16–19% in backs and forwards, respectively.

### Speed and Change of Direction Speed

Linear and change of direction speed are important physical qualities for RU and are associated with line breaks, evading and beating defenders and metres advanced in senior players [72]. Six studies are available within age-grade England RU players [46, 61–65] presenting initial (i.e. 5 and 10 m), maximal sprinting (i.e. 30 and 40 m) and sprint momentum data. However, only one study presents a change of direction speed via the 505 test [62]. Age does not differentiate between initial [61, 62] or 20-m speed except in forwards [63], in whom it was increased in older age categories. Forty-metre speed was superior at older age categories in backs and forwards [62, 63]. Initial [62, 64] and maximal [63] sprint momentum increased with age suggesting this should be measured and tracked. Change of direction speed was also greater at U21 age categories compared to U16 and U18 [62].

Backs were faster than forwards across initial and maximal sprint distances [63]. Academy level players outperformed aged-matched school players for 20-m speed when compared across studies [46, 62], although small differences were only apparent at 40 m and for sprint momentum in direct comparisons [65]. When compared to other studies, English age-grade RU players are slower than South African U20 internationals [59] and professional players [73, 74].

### Aerobic Capacity

Enhanced aerobic capacity is important for RU due to the need to recover quickly from high-intensity efforts [75]. Five studies present the aerobic capacity qualities of age-grade RU players using the Yo-Yo intermittent recovery level 1 [62, 63, 65], 30–15 intermittent fitness [60, 62, 63] and a laboratory-based VO_2_max [49] test. Small differences in aerobic capacity, which were greater in older age categories, were shown [62, 63], and these differences increase when body mass is accounted for within the statistical analysis [60]. Body mass should therefore be considered when measuring and tracking aerobic capacity in youth players. Comparisons between playing position and standard demonstrate backs generally have greater aerobic capacity than forwards [63] consistent with other research [58, 59] and academy players outperform school players [65].

### Muscular Strength and Power

Muscular strength and power are key attributes of RU performance due to the contact and collision element of the sport [75]. Six studies have presented strength and power data in age-grade RU players via Wattbike peak power output [64], countermovement jump [46, 62] or isoinertial strength tests [46, 62, 65–67]. Strength and power are greater in older age categories [62, 64] supporting data in rugby league [53, 68]. Furthermore, strength and power differentiate between playing standard [65] and resistance training experience [67].

### Summary

Overall, physical qualities increase with age and playing level and differ between forwards and backs demonstrating the importance of their development in age-grade RU players. However, current evidence and normative data are limited by studies only utilising one club. Future research should aim to develop and implement a national standardised fitness testing battery allowing the quantification of the physical qualities of age-grade RU players throughout England for talent identification, player monitoring and development. A further focus on players aged below 16 years is required, whilst considering maturity status, alongside implementing longitudinal research designs [76, 77] and considering the importance of physical qualities for match performance and long-term career outcomes.

## Fatigue and Recovery

Understanding the fatigue and recovery profiles of RU players following training and match-play provides important information for planning appropriate training and competition schedules [78]. Studies within senior RU players have demonstrated that post-match fatigue may manifest as acute reductions in neuromuscular function [79, 80], elevations in markers of muscle damage [81, 82], alterations in immune and endocrine function [79, 83, 84] and negative changes in mood [79, 80] up to 60-h post match-play. In addition to studies conducted in other youth RU populations [85, 86], six studies were identified investigating fatigue markers post match-play [32, 87] and training [50, 88–90] within male age-grade RU players from England (Table 6).

**Table 6:** Fatigue and recovery profiles post match-play and training in male youth rugby union players from England

Study	Sample level	Training/match-play	Measures	Results
Johnston et al. [89]	U21 academy (*n* = 15)	Training (comparison of speed-weights vs. weights-speed training order)	CMJ, muscle soreness, blood lactate, CK, testosterone and cortisol	Speed-weights CMJ − Pre = 0.40 ± 0.05, 24 h = 0.37 ± 0.06 m; CK − Pre = 485 ± 420, 24 h = 1161 ± 816 u/l; testosterone − Pre = 16.3 ± 3.7, 24 h = 17.4 ± 4.0 mmol/l; cortisol − Pre = 491 ± 103, 24 h = 520 ± 106 mmol/l; lactate − Pre = 1.50 ± 0.72, 24 h = 0.89 ± 0.49 mmol/l; soreness = 1.7 ± 0.8, 24 h = 3.8 ± 1.2 AU Weights-speed CMJ − Pre = 0.39 ± 0.06, 24 h = 0.37 ± 0.06 m; CK − Pre = 508 ± 306, 24 h = 1122 ± 946 u/l; testosterone − Pre = 17.1 ± 4.9, 24 h = 17.7 ± 4.6 mmol/l; cortisol − Pre = 516 ± 199, 24 h = 514 ± 100 mmol/l; lactate − Pre = 1.25 ± 0.66, 24 h = 1.31 ± 0.77 mmol/l; soreness = 1.9 ± 0.9, 24 h = 3.7 ± 1.1 AU Sig (*p* < 0.05) time effects but no time vs. protocol interactions found
Noon et al. [90]	U18 college (*n* = 10)	Training (comparison of low vs. high training volume)	CMJ, well-being, resting HR, HRV	Motivation (AU)—low = − 0.7 ± 1.7, high = −1.9 ± 1.9; sleep quality (AU)—low = 0.3 ± 1.1, high = − 1.0 ± 1.1; recovery (AU)—low = − 0.2 ± 1.7, high = − 2.4 ± 1.8; appetite (AU)—low = 0.0 ± 1.7, high = 0.7 ± 0.9; fatigue (AU)—low = 0.2 ± 1.6, high = 0.9 ± 1.6; stress (AU)—low = 0.2 ± 0.2, high = 0.6 ± 1.6; muscle soreness (AU)—low = 1.1 ± 1.5, high = 2.0 ± 1.7; CMJ—low = 37.2 ± 4.4, high = 37.2 ± 4.4 cm; rest HR—low = 58 ± 1, high = 64 ± 4 bpm; in SDNN—low = 1.96 ± 0.09, high = 1.88 ± 0.13; in rMSSD—low = 1.94 ± 0.18, high = 1.81 ± 0.18
Roe et al. [87]	U18 academy (*n* = 14)	Match-play	Adductor strength	Immediately = − 1.3 ± 2.5 %; ES = − 0.11 ± 0.21; 24 h = − 0.7 ± 3%; ES = − 0.06 ± 0.25; 48 h = 3.8 ± 1.9%, ES = 0.32 ± 0.16; 72 h = 3.1 ± 2.2%, ES = 0.26 ± 0.18
Roe et al. [32]	U18 academy (*n* = 14)	Match-play	CMJ, PPU, plasma CK and perception of well-being	CMJ mean power immediately = − 5.5 ± 3.3%, 24 h = − 7.0 ± 3.9 %, 48 h = − 5.8 ± 5.4 %, 72 h = − 0.8 ± 3.8 %; PPU flight-time—immediately = − 15.3 ± 7.3%, 24 h = − 11.5 ± 5.7%, 48 h = 3.5 ± 6%, 72 h = − 0.9 ± 5.4%; well-being—24 h = − 24 ± 4.3%, 48 h = − 8.3 ± 5.9%, 72 h = − 3.6 ± 3.7%; CK—immediately = 138.5 ± 33.1%, 24 h = 326 ± 77.6%, 48 h = 176.4 ± 62.4%, 72 h = 56.7 ± 34.5%
Roe et al. [88]	U18 academy (*n* = 20)	Training (contact vs. no contact training)	CMJ, PPU, CK, 6-item wellbeing	CMJ mean power—24 h post contact = − 2.3 ± 2.4 %, 24 h post non-contact = − 5.4 ± 5.2 % (*possibly* greater non-contact); PPU flight time—24 h post contact = − 7.3 ± 24.7 %, 24 h post non-contact = 2.7 ± 5.9 % (v*ery likely* greater contact); CK—24 h post contact = 88.2 ± 40.7 %, 24 h post non-contact = 0 % (*almost certainly* greater contact); wellbeing—24 h post contact = − 8.0 ± 4.8 %, 24 h post non-contact = − 3.4 ± 2.2 % (*likely* greater contact)
Roe et al. [50]	U20 academy (*n* = 14)	Training (pre-season changes)	CMJ flight time, mean power and mean force, maximum velocity and 3RM front squat	CMJ mean power—likely, very likely or almost certain reductions at week 2 and 5 to 11 CMJ flight time—likely, very likely or almost certain Reductions at week 2, 4 to 6 and 9 to 10. CMJ mean force—all findings trivial 40 m maximum velocity—very likely improvements in 40 m sprint velocity (5.5 ± 3.6%) occurred between week 1 and week 10 3RM front squat—possible improvements in lower body strength (5.8 ± 2.7%) were made from week 1 to week 10,

*U* under, *CMJ* countermovement jump, *CK* creatine kinase, *HR* heart rate, *HRV* heart rate variability, i*n rMSSD* root square of the mean squared differences of successive R-R intervals, *in SDNN* natural logarithm of the standard deviation of R-R intervals, *PPU* plyometric push up, *ES* effect size

### Match-Play Fatigue

Fatigue and recovery post academy RU match-play has been assessed using the adductor squeeze [87], markers of lower-body (countermovement jump) and upper-body (plyometric push-up) neuromuscular function, subjective assessment of wellness and proxy methods of skeletal muscle damage (e.g. creatine kinase concentrations [CK]) [32]. With the exception of adductor squeeze, which showed trivial reductions in response to match-play [87], markers of neuromuscular function, wellness and muscle damage all demonstrated peak changes in the first 24 h post-match [32]. Lower-body neuromuscular function remained substantially reduced at 48 h post-match, whilst both CK and wellness were still substantially altered at 72 h following match-play, although recovering at this time [32]. Such findings are consistent with findings in senior RU and age-grade RU players [85, 86] and other youth sports (e.g. rugby league, [91]; Australian Football [78]). These findings suggest that young RU players should be afforded a minimum of one recovery day (i.e. active or passive) following competition before returning to training at 48 h post-match. However, practitioners should aim to monitor player recovery on an individual basis due to the large inter-individual responses to match-play reported, in order to appropriately plan individualised training schedules. Unfortunately such practice is not always adopted in English age-grade RU, based on the reported training and match practices [13, 48].

### Training Fatigue

Four studies have evaluated fatigue and recovery responses to training, considering training volume [90], session order [89], contact training [88] and longitudinal responses over an 11-week pre-season period [50]. All studies have demonstrated a fatigue response to training with the magnitude of response-dependent upon the training undertaken. For example, Noon et al. [90] demonstrated greater perceptions of fatigue following high vs. low training volume. Roe et al. [88] demonstrated substantially greater upper body neuromuscular fatigue, a decrease in wellness and elevated CK following contact training, whilst lower body neuromuscular fatigue was substantially increased following non-contact training, indicative of the greater running volumes and intensities. Session order [89] did not affect fatigue responses post speed-weights or weights-speed training (i.e. 6 × 50 m sprints with 5 min recovery; 5 sets × 4 repetitions at 85% 1RM with 4 min rest of back squat and Romanian deadlift). However, speed was enhanced when this was performed following 1.76 ± 0.08 s rather than prior to a weights session (1.80 ± 0.11 s) possibly due to a post-activation potentiation effect [89]. During an 11-week pre-season, lower-body neuromuscular fatigue was present throughout the majority of the observational period, however, was greatest during the periods of higher training volume. Despite this, improvements in 3RM front squat strength and maximum sprint velocity were observed, suggesting enhancements in physical performance can still be achieved when fatigue, as measured by a countermovement jump, is present [50]. These findings provide a challenge to all practitioners in planning appropriate training to prepare players for weekly match-play whilst still maintaining a long-term athlete development focus.

### Summary

Overall, fatigue is present in age-grade RU players following match-play and training. Following U18 academy RU match-play, peak changes in markers of fatigue are seen in the first 24 h, with some taking more than 72 h to return to baseline levels. Furthermore, fatigue responses following training can be affected by training volume, activities (e.g. contact) and session order. These factors are further confounded by the large inter-individual fatigue responses following match-play and training. Such findings provide an interesting challenge to practitioners in planning and delivering training schedules. Practitioners should aim to provide a minimum of one recovery day (e.g. active or passive) following competition before returning to training at 48 h post-match and monitor player recovery on an individual basis where possible. Future research should explore the consequences of changes in measures of fatigue (i.e. injury, reductions in performance) whilst exploring fatigue responses following combined match-play and training schedules over longitudinal periods.

## Nutritional Requirements

Performance nutrition is another key aspect of supporting the adaptations to training and match-play alongside maintaining appropriate growth and health of the age-grade RU player [92]. Whilst research exists exploring the nutritional requirements, intakes and expenditures of adult male players [51, 93, 94], there are only two studies that exist within English male age-grade players [95, 96].

### Energy Requirements

Using doubly labelled water, the mean total energy expenditure of 14 English age-grade players was 4369 ± 979 kcal day^−1^ [96], suggesting higher energy expenditure (approximately 500 kcal day^−1^) than estimated via traditional equations (e.g. Harris-Benedict [97]). Increased energy expenditures may be apparent due to the metabolic cost of the collision identified in youth rugby league [98].

### Energy Intakes

Only one study to date [95] has assessed the energy intakes of age-grade RU players. Using a 4-day food diary, mean energy intake for the U16 players was 3269 ± 766 kcal·day^−1^, with protein and carbohydrate intakes reported relative to body mass as 1.9 ± 0.6 and 4.8 ± 1.1 g kg^−1^ day^−1^, respectively. For the U19 players (*n* = 21), their mean energy intake was 3412 ± 670 kcal day^−1^ whilst mean protein was 2.3 ± 0.5 g kg^−1^ day^−1^ and mean carbohydrate intake was 4.7 ± 1.4 g kg^−1^ day^−1^. Energy intakes were lower than reported energy expenditure values [96], although the players met the standard guidelines for energy and macronutrients. This is similar to data from Australian rugby players of the same age [99].

### Micronutrient Requirements

To date, there are no published data in English RU age-grade players to guide specific micronutrient requirements or their corresponding dietary intakes and therefore the standard healthy guidelines should be used. When considering dietary quality (and its correlation to micronutrient intakes), Smith et al. [95] showed that U19 players achieved the recommended servings of fruit and vegetables per day, whilst U16 players did not within Yorkshire, England.

### Summary

Research exploring the energy intakes and expenditure of age-grade RU players is limited. In the absence of specific data, sports nutrition guidelines for adults can be used in combination with nutrition periodisation and frequent monitoring. Practically, dietary intake assessments, using novel methods (e.g. Snap-and-Send [100]) along with serial measurements of growth, physiological development, strength and self-reported fatigue and recovery may be an optimal combination to assist with adapting a standard nutritional prescription rather than the use of any static targets.

## Psychological Challenges and Development

Psychology is acknowledged as a key determinant in the realisation of potential and long-term success in sport [101], especially RU [102]. However, despite this importance, the prevalence of systematic psychological inquiry into both senior and youth populations worldwide in the sport is scarce. To date, five studies have investigated the psychological challenges and developmental demands faced by age-grade English RU players. These studies have focused upon the stress and coping experiences of players [103–105] and the psychological factors contributing to successful talent development [102, 106] (Table 7).

**Table 7:** Psychological challenges and development in male youth rugby union players from England

Study	Sample	Level	Measures	Results
Nicholls and Polman [105]	11	U18 national squad	Stressor checklist, coping responses, perceived coping effectiveness	Most frequently-cited stressors: making a mental or physical error, receiving coach/parental criticism, and injury; coping strategies: blocking, increasing effort, and taking advice: blocking and technical adjustment rated as more effective strategies
Madigan et al. [104]	13	Further education Academy athletes	Perfectionism, injury	Perfectionism positively predicted injury; only perfectionistic concerns emerged as a significant positive predictor; likelihood of sustaining injury increased twofold for each 1 SD increase in perfectionistic concerns
Hill and Appleton [103]	202	U19 youth	Athlete burnout, multidimensional perfectionism, perfectionistic cognitions	Frequency of perfectionistic cognitions positively related to all symptoms of athlete burnout; frequency of perfectionistic cognitions explained 3–4% unique variance in symptoms of athlete burnout after controlling for self-oriented and socially prescribed dimensions of perfectionism
Hill et al. [102]	15	Premiership academy directors and head coaches	Interview guide explored psychological aspects that may facilitate or derail talent development processes positive	Positive psychological characteristics: cognitive ability, competitiveness, confidence and self-belief, consistency, courage, cultural identity, developmental awareness, driving group standards, effective communication, emotional intelligence, flexibility and adaptability, game understanding, grit. Dual-effect psychological characteristics: aggression, obsessive passion, over-commitment, over-confidence, perfectionism, preestablished frameworks and beliefs, work-life balance. Negative psychological characteristics: avoidance-based coping strategies, complacency, disorganised, expectation and entitlement, failure to overcome challenge, inappropriate goals, lack of awareness, lack of commitment, loss of focus/easily distracted, mental health, negative attitude, poor communicators, psychological burnout, self-doubt, self-handicapping, shyness.
McCarthy et al. [106]	821	U18 academy	Player birth month distribution	Skewed birth date distribution across quartiles between observed and expected values; clear bias with Q1 (*n* = 336, 41%) and Q2 (*n* = 175, 22%), different to Q3 (*n* = 176, 21%) and Q4 (*n* = 134, 16%)

*Q* quartile (Q1 = September to November, Q2 = December to February, Q3 = March to May, Q4 = June to August).

### Stress and Coping Experiences

Nicholls and Polman [105] examined the stressors, coping strategies and perceived coping effectiveness amongst England U18 international RU players. The most frequently cited stressors were making a mental or physical error, receiving coach/parental criticism and injury. Coping strategies included blocking, increasing effort and taking advice, with blocking and technical adjustment strategies rated as being more effective. Two studies have also considered the impact of the personality variable of perfectionism upon physical and mental health symptoms. According to the stress-injury model [107], personality factors which predispose athletes to elevated levels of stress (e.g. perfectionism) may increase the risk of injury. A prospective study examined the perfectionistic strivings, perfectionistic concerns and injury [104] showing whilst perfectionism positively predicted injury, only perfectionistic concerns emerged as a significant positive predictor. Perfectionism, and the frequency of the experience of perfectionistic cognitions, has also been identified as a psychological trait which is an antecedent of athlete burnout and a precursor to sport dropout. An investigation of male RU players from youth teams [103] reported frequency of perfectionistic cognitions explained 3–4% variance in symptoms of athlete burnout after controlling for self-oriented and socially prescribed dimensions of perfectionism. Collectively, these findings suggest that individuals who have perfectionistic concerns are at a greater risk of injury. In addition, the frequency with which perfectionistic cognitions are experienced may also be an antecedent of athlete burnout. Perfectionistic cognitions should, therefore, be considered in future models of the relationship between perfectionism, injury and athlete burnout.

### Psychological Factors Contributing to Successful Talent Development

Acknowledging psychology in providing important information for talent identification and successful development to the elite level, Hill et al. [102] interviewed English RU academy coaches and directors to identify the positive and negative issues influencing talent development. Whilst support was found for a range of positive constructs (e.g. planning and self-organisation, commitment and resilience) as facilitators of effective development, negative and dual (inappropriately applied ‘positive’) characteristics (e.g. obsessive passion and perfectionism) had a negative impact on development. One concept highlighted extensively within the sports science literature as influencing talent selection and identification within sports is the relative age effect (RAE). McCarthy et al. [106, 108] investigated this initial bias in professional RU academies and found a reversal of the RAE effect, whereby relatively young players were less likely to be selected into their respective national academy systems but more likely to transition into senior professional squads. The role of adversity in promoting growth and flourishing was suggested as a psychological explanation for such an effect, with exposure to adversity considered as an element of a successful talent system.

### Summary

RU players face a range of psychological demands and adopt numerous strategies to cope with these challenges. Perfectionistic cognitions are a potential factor predisposing young players to an increased injury risk and should be considered when designing interventions to reduce perfectionism and burnout. Understanding the psychological characteristics that facilitate and derail progression can enhance coaches’ player assessment when identifying and supporting youth RU players. Given the limited literature to date, future research should seek to examine in greater depth the psychological demands age-grade RU players from England face, the skills/strategies deployed to successfully transition to the elite professional level and the factors (e.g. personal, situational, organisational and cultural) that mediate this progression.

## Injury

Injury risk across RU has drawn public and academic interest, with concerns that the associated injury risk is high at youth levels coinciding with calls to modify the game by removing playing events such as the tackle [109–111]. Descriptive epidemiological studies of injury patterns are regarded as a foundation from which potential injury risk factors can be identified and preventive strategies formulated [112]. Studies describing injury patterns exist within senior elite [113–115], senior community [116–118], academy [45, 119] and youth community [45, 119–121] RU players within England. Within age-grade RU specifically, four studies described injury patterns within England [45, 119–121], and one further study investigated the efficacy of a preventive measure [122]. Table 8 summarises the key findings of descriptive epidemiological studies in English age-grade RU.

**Table 8:** Summary of descriptive epidemiological studies of injuries conducted in youth rugby union players from England

Study	Study length	Sample level	Number of players	Number of exposure hours	Number of injuries	Results
Match injury—greater than 24-h time-loss						
Haseler et al. [120]	1 playing season (9 months)	U9 to U17 Club	210	1636 player-match hours	39	Overall match injury incidence—24/1000 player-match hours Mean severity—32 days By injury location: head/neck—26%; upper limb—31%; trunk—10%; lower limb—33%
Palmer-Green et al. [119]	2 playing seasons	U18 elite schoolboy	222	3843 player-match hours	134	Overall match injury incidence—35/1000 player-match hours Mean severity—27 days By injury location: head/neck—18%; upper limb—24%; trunk—10%; lower limb—47%
	2 playing seasons	U18 academy	250	2343 player-match hours	109	Overall match injury incidence—47/1000 player-match hours Mean severity—33 days By injury location: head/neck—14%; upper limb—28%; trunk—3%; lower limb—55%
Barden and Stokes [121]	3 playing seasons	U18 elite schoolboy	132	595 player-match hours	46	Overall match injury incidence—77/1000 player-match hours Mean severity—20 days By injury location: head/neck—32%; upper limb—32%; trunk—10%; lower limb—25%
		U18 sub-elite schoolboy		1698 player-match hours	57	Overall match injury incidence—34/1000 player-match hours Mean severity—19 days By injury location: head/neck—15%; upper limb—26%; trunk—6%; lower limb—53%
Training injury—greater than 24-h time-loss						
Palmer-Green et al. [45]	2 playing seasons	U18 elite schoolboy	222	15,877 player-training hours	34	Overall training injury incidence—2.1/1000 player-training hours Mean severity—27 days By injury location: head/neck—9%; upper limb—15%; trunk—32%; lower limb—44%
	2 playing seasons	U18 academy	250	47,431 player-training hours	64	Overall training injury incidence—1.4/1000 player-training hours Mean severity—17 days By injury location: head/neck—9%; upper limb—13%; trunk—13%; lower limb—65%

### Injury Risk

Within age-grade RU in England, match injury rates ranged between 24 and 77 injuries per 1000 player-match hours (using a greater than 24-h time-loss injury definition) [123, 124]. These injury rates broadly correspond with documented match injury rates (using a comparable injury definition) from male age-grade RU in Northern Ireland (ages 16–18 years, 29/1000 player-match hours [123]) and South Africa (ages 12–18 years, 20/1000 player-match hours [124]). Match injury rates from English age-grade rugby also largely fall within the range outlined in the findings of a meta-analysis across both RU and rugby league in children and adolescent players (aged < 21 years) from a range of settings that revealed a pooled overall match injury incidence rate of 27 injuries/1000 player-match hours (95% confidence limits 13–54), irrespective of injury definition [110]. The lower limb has been shown to be the most frequently injured body location in age-grade RU players, accounting for 33–55% of all match injuries, followed by the upper limb (24–32%), head/neck region (14–32%) and trunk (3–10%) [119–121]. Additionally, joint and ligament injuries are commonly reported injury types amongst young RU players (39–51%), followed by musculotendinous injuries (18–24%), lacerations/contusions (18–19%) and bone fractures (6–8%) [119]. The knee and shoulder joints have been shown to be at a particularly high risk of severe injuries such as ligament injuries (sprains), fractures and dislocations [119], whilst concussion has recently been recognised amongst the most common and severe injury diagnoses experienced by male age-grade RU players [121]. The tackle situation is the most commonly recorded match event associated with injury and accounts for 51–57% of match injuries [119, 121]. In contrast to match injury rates, the limited amount of evidence relating to training injuries in English age-grade RU slows rates to be much lower at between 1.4 and 2.1 injuries per 1000 player-training hours [45].

Whilst acknowledging the limited number of studies at present, data from included studies represent a limited number of settings, namely U18 male players in academies or schools. Consequently, the nature and pattern of injuries experienced by other RU-playing populations is uncertain, particularly within youth and children and community club rugby.

### Prevention Strategies

A number of approaches have been reported to positively affect injury risk across RU, including law alterations [125, 126], coach and referee education [127–130] and protective equipment [131, 132]. Whilst these preventive measures can readily apply to English youth players, only one study has been conducted to directly assess the efficacy of preventive measures. A recent study in schoolboy RU players (aged 15–18 years) revealed that a targeted pre-activity preventive exercise programme over one playing season (August to December) containing balance and bodyweight resistance exercises reduced measures of upper limb injury (by 34%) and concussion (by 29%) amongst players when compared with a standard of practice (control) exercise programme [122]. The mechanisms underlying the observed reductions are unclear but may relate to training effects on joint kinematics and force-handling capabilities in the upper body [133, 134], whilst developing or preserving aspects of neck function, such as strength, may have contributed to the reduction in concussion incidence [135, 136]. Furthermore, when comparisons were made across teams that regularly used the respective exercise programmes three or more times per week, those assigned to the targeted preventive exercise programme suffered 72% fewer match injuries when compared with the control exercise programme, with a noticeable reduction of 59% in concussion risk [122]. This suggests targeted preventive exercise programmes may be effective as injury prevention methods.

### Summary

Despite a limited number of studies at present, documented injury patterns in English age-grade RU appear similar to other RU populations. The relatively high incidence of soft tissue injuries and concussion in this population highlights a need to focus on reducing the risk of these priority injury types. Recent evidence supports including targeted preventive exercise programmes into age-grade RU as a means of reducing soft-tissue injury and concussion risk. Future research should explore the exact nature of injuries arising from prominent match events such as the tackle situation, which may inform strategies to reduce injury risk in age-grade RU players.

## Conclusions

International associations (e.g. International Olympic Committee) and national governing bodies (e.g. RFU) have emphasised the importance of designing and implementing healthy youth athletic development programmes. Within RU in England, this is even more important due to the employment of a late specialisation sporting system resulting in a complex multi-sport, multi-environment and multi-coach development programme. Although such a system has potential benefits, it also challenges the optimisation and maintenance of health, participation and player development within RU in England. This review provides a first attempt to present current evidence on the applied sport science of male age-grade RU players within England and summarises and critically appraises the literature in relation to the (1) match-play characteristics, (2) training exposures, (3) physical qualities, (4) fatigue and recovery, (5) nutrition, (6) psychological challenges and development and (7) injury.

Current evidence suggests that match-play characteristics are influenced by age, playing level and position. However, no information is available considering the technical and tactical elements of match-play that are common within the adult game [137–139] and is therefore a future research direction. Youth players’ weekly and monthly training exposure represents a highly variable structure with reduced week to week stability due to potential misalignment of fixtures, which may cause potential negative outcomes (e.g. injury). Alongside this, considering it can take 72 h for fatigue markers to return to baseline post-match means consideration of training and competition frequency, volume and intensity are important for maximising positive and negative responses. The training exposure and physical qualities of players increase with age and playing level and differ between forwards and backs. However, it could be questioned whether appropriate strategies (e.g. training load variability and training modality gym exposure) are implemented to maximise player development. In addition to physical factors, the psychological challenges and development facing age-grade RU players are widespread with the evidence base alluding to perfectionism and burnout as two major factors potentially predisposing players to injury. Finally, injury risk and the energy demands of young players are high and therefore require careful consideration within practice.

Based on the above, all coaches, administrators and stakeholders should consider the applied sport science and research evidence base in the appropriate and healthy development of age-grade RU athletes. This includes appropriate scheduling and inclusion of training and match-play activities that aim to maximise athlete development (e.g. physical qualities) whilst reducing and minimising the negative consequences (e.g. injury and burnout). Through the planning and delivery of age-grade RU training, players should be provided a minimum of one rest day (active or passive) post competition with players ideally monitored on an individual basis. Further exposing players to structured sprint training, resistance training within a microcycle, the management of training and competition exposure and the assessment of potential psychological behaviours (e.g. perfectionism) should be high priorities. Recent interventions implemented by the RFU including the half-game rule [140] may help achieve this aim whilst ensuring both participation and player development opportunities.

Although the current evidence base is emerging, most studies are limited by the inclusion of only one club, potentially challenging the reach of the findings. The implementation of national research projects including standardised fitness testing, load and recovery monitoring and injury audits may enhance the understanding and evaluation of programmes for ensuring healthy athletic development. Furthermore, research exploring the interactions and integration between match-play characteristics, training load, physical qualities, fatigue recovery and injury would be deemed important rather than evaluation within isolation. A greater focus upon the psychological and holistic developmental needs of age-grade players (e.g. nutrition, illness, maturity, technical and tactical performance) is a direction for future research that would inform coach and stakeholder education within RU.

## Data Availability

Not applicable

## Abbreviations

AU: Arbitrary units
CK: Creatine kinase
CV: Coefficient of variation
GPS: Global Positioning Systems
PL: PlayerLoad
PL_slow_: PlayerLoad slow
RAE: Relative age effect
RFU: Rugby Football Union
RU: Rugby union
sRPE: Session rating of perceived exertion

## References

[1] World Rugby. Global Rugby Participation. 2016. https://www.worldrugby.org/development/player-numbers?lang=en. Accessed 22 Mar 2019.

[2] Hendricks S, van Niekerk T, Sin DW, Lambert M, den Hollander S, Brown J (2017). Technical determinants of tackle and ruck performance in international rugby union. J Sports Sci.

[3] Read DB, Jones B, Phibbs PJ, Roe GA, Darrall-Jones JD, Weakley JJ (2017). Physical demands of representative match-play in adolescent rugby union. J Strength Cond Res.

[4] McLaren SJ, Weston M, Smith A, Cramb R, Portas MD (2016). Variability of physical performance and player match loads in professional rugby union. J Sci Med Sport.

[5] Read D, Weaving D, Phibbs P, Darrall-Jones J, Roe G, Weakley J (2017). Movement and physical demands of school and university rugby union match-play in England. BMJ open Sport Ex Med.

[6] Duthie G, Pyne D, Hooper S (2003). Applied physiology and game analysis of rugby union. Sports Med.

[7] Pollard BT, Turner AN, Eager R, Cunningham DJ, Cook CJ, Hogben P (2018). The ball in play demands of international rugby union. J Sci Med Sport.

[8] Freitag A, Kirkwood G, Pollock AM (2015). Rugby injury surveillance and prevention programmes: are they effective?. BMJ..

[9] Phibbs PJ, Jones B, Read DB, Roe GA, Darrall-Jones J, Weakley JJ (2018). The appropriateness of training exposures for match-play preparation in adolescent schoolboy and academy rugby union players. J Sports Sci.

[10] Hendricks S, Till K, Weaving D, Powell A, Kemp S, Stokes K (2019). Training, match and non-rugby activities in elite male youth rugby union players in England. Int J Sports Sci Coach.

[11] Côté J, Vierimaa M (2014). The developmental model of sport participation: 15 years after its first conceptualization. Sci Sports.

[12] Noon MR, James RS, Clarke ND, Akubat I, Thake CD (2015). Perceptions of well-being and physical performance in English elite youth footballers across a season. J Sports Sci.

[13] Phibbs PJ, Jones B, Roe G, Read D, Darrall-Jones J, Weakley J (2018). The organised chaos of English adolescent rugby union: influence of weekly match frequency on the variability of match and training loads. Eur J Sport Sci.

[14] Lloyd RS, Oliver JL, Faigenbaum AD, Howard R, Croix MBDS, Williams CA (2015). Long-term athletic development-part 1: a pathway for all youth. J Strength Cond Res.

[15] Lloyd RS, Cronin JB, Faigenbaum AD, Haff GG, Howard R, Kraemer WJ (2016). National Strength and Conditioning Association position statement on long-term athletic development. J Strength Cond Res.

[16] Bergeron MF, Mountjoy M, Armstrong N, Chia M, Côté J, Emery CA (2015). International Olympic Committee consensus statement on youth athletic development. Br J Sports Med.

[17] England Rugby. Age grade rugby. Available from: https://www.englandrugby.com/my-rugby/players/age-grade-rugby/about-age-grade-rugby/. 2017. Accessed 22 June 2019

[18] McGuigan MM. Extreme positions in sport science and the importance of context: it depends? 2016; 11(7):841 10.1123/IJSPP.2016-0513 27869557

[19] Lambert MI, Durandt J (2010). Long-term player development in rugby–how are we doing in South Africa?. S Afr J Sports Med.

[20] Cummins C, Orr R, O’Connor H, West C (2013). Global positioning systems (GPS) and microtechnology sensors in team sports: a systematic review. Sports Med.

[21] Austin D, Gabbett T, Jenkins D (2011). The physical demands of Super 14 rugby union. J Sci Med Sport.

[22] Cahill N, Lamb K, Worsfold P, Headey R, Murray S (2013). The movement characteristics of English Premiership rugby union players. J Sports Sci.

[23] Quarrie KL, Hopkins WG, Anthony MJ, Gill ND (2013). Positional demands of international rugby union: evaluation of player actions and movements. J Sci Med Sport.

[24] Roberts SP, Trewartha G, Higgitt RJ, El-Abd J, Stokes KA (2008). The physical demands of elite English rugby union. J Sports Sci.

[25] Deutsch M, Maw G, Jenkins D, Reaburn P (1998). Heart rate, blood lactate and kinematic data of elite colts (under-19) rugby union players during competition. J Sports Sci.

[26] Hartwig TB, Naughton G, Searl J (2011). Motion analyses of adolescent rugby union players: a comparison of training and game demands. J Strength Cond Res.

[27] Portillo J, González-Ravé JM, Juárez D, García JM, Suárez-Arrones L, Newton RU (2014). Comparison of running characteristics and heart rate response of international and national female rugby sevens players during competitive matches. J Strength Cond Res Research.

[28] Venter RE, Opperman E, Opperman S (2011). The use of Global Positioning System (GPS) tracking devices to assess movement demands and impacts in Under-19 rugby union match play. Afr J Phys Health Educ Recreat Dance.

[29] Flanagan EPL, Lacome M (2017). The demands of the game – a descriptive analysis of the locomotor demands of Junior International Rugby Union. J Aust Strength Cond.

[30] Read DB, Jones B, Phibbs PJ, Roe GA, Darrall-Jones J, Weakley JJ (2018). The physical characteristics of match-play in English schoolboy and academy rugby union. J Sports Sci.

[31] Read DB, Till K, Beasley G, Clarkson M, Heyworth R, Lee J (2019). Maximum running intensities during English academy rugby union match-play. Sci Med Football.

[32] Roe G, Till K, Darrall-Jones J, Phibbs P, Weakley J, Read D (2016). Changes in markers of fatigue following a competitive match in elite academy rugby union players. South African Journal of Sports Medicine.

[33] Roe G, Halkier M, Beggs C, Till K, Jones B (2016). The use of accelerometers to quantify collisions and running demands of rugby union match-play. Int J Perform Anal Sport.

[34] Read DB, Jones B, Williams S, Phibbs PJ, Darrall-Jones JD, Roe GA (2018). The physical characteristics of specific phases of play during rugby union match play. Int J Sports Physiol Perform.

[35] Cunningham D, Shearer DA, Drawer S, Eager R, Taylor N, Cook C (2016). Movement demands of elite U20 international rugby union players. PLoS One.

[36] Gabbett TJ, Jenkins DG, Abernethy B (2011). Relationships between physiological, anthropometric, and skill qualities and playing performance in professional rugby league players. J Sports Sci.

[37] Whitehead S, Till K, Weaving D, Jones B (2018). The use of microtechnology to quantify the peak match demands of the football codes: a systematic review. Sports Med.

[38] Cunningham DJ, Shearer DA, Carter N, Drawer S, Pollard B, Bennett M (2018). Assessing worst case scenarios in movement demands derived from global positioning systems during international rugby union matches: rolling averages versus fixed length epochs. PLoS One.

[39] Gabbett TJ, Nassis GP, Oetter E, Pretorius J, Johnston N, Medina D (2017). The athlete monitoring cycle: a practical guide to interpreting and applying training monitoring data. Br J Sports Med.

[40] Hartwig TB, Naughton G, Searl J (2008). Defining the volume and intensity of sport participation in adolescent rugby union players. Int J Sports Physiol Perform.

[41] Hartwig TB, Naughton G, Searl J (2009). Load, stress, and recovery in adolescent rugby union players during a competitive season. J Sports Sci.

[42] Gabbett TJ, Whyte DG, Hartwig TB, Wescombe H, Naughton GA (2014). The relationship between workloads, physical performance, injury and illness in adolescent male football players. Sports Med.

[43] Lacome M, Carling C, Hager J-P, Dine G, Piscione J (2018). Workload, fatigue, and muscle damage in an under-20 rugby union team over an intensified international tournament. Int J Sports Physiol Perform.

[44] Quarrie KL, Raftery M, Blackie J, Cook CJ, Fuller CW, Gabbett TJ (2017). Managing player load in professional rugby union: a review of current knowledge and practices. Br J Sports Med.

[45] Palmer-Green DS, Stokes KA, Fuller CW, England M, Kemp SP, Trewartha G (2015). Training activities and injuries in English youth academy and schools rugby union. Am J Sports Med.

[46] Weakley JJS, Till K, Darrall-Jones J, Roe GAB, Phibbs PJ, Read DB (2019). Strength and conditioning practices in adolescent rugby players: relationship with changes in physical qualities. J Strength Cond Res.

[47] Phibbs PJ, Jones B, Roe GA, Read DB, Darrall-Jones J, Weakley JJ (2017). We know they train, but what do they do? Implications for coaches working with adolescent rugby union players. Int J of Sports Coach.

[48] Phibbs PJ, Jones B, Roe G, Read DB, Darrall-Jones J, Weakley J (2018). Organized Chaos in late specialization team sports: weekly training loads of elite adolescent rugby union players. J Strength Cond Res.

[49] Taylor RJ, Sanders D, Myers T, Abt G, Taylor CA, Akubat I (2018). The dose-response relationship between training load and aerobic fitness in academy rugby union players. Int J Sports Physiol Perform.

[50] Roe GA, Darrall-Jones JD, Till K, Jones B (2016). Preseason changes in markers of lower body fatigue and performance in young professional rugby union players. Eur J Sport Sci.

[51] Bradley WJ, Cavanagh BP, Douglas W, Donovan TF, Morton JP, Close GL (2015). Quantification of training load, energy intake, and physiological adaptations during a rugby preseason: a case study from an elite European rugby union squad. J Strength Cond Res.

[52] Cross MJ, Williams S, Trewartha G, Kemp SP, Stokes KA (2016). The influence of in-season training loads on injury risk in professional rugby union. Int J Sports Physiol Perform.

[53] Argus CK, Gill ND, Keogh JW (2012). Characterization of the differences in strength and power between different levels of competition in rugby union athletes. J Strength Cond Res.

[54] Smart DJ, Hopkins WG, Gill ND (2013). Differences and changes in the physical characteristics of professional and amateur rugby union players. J Strength Cond Res.

[55] Crewther B, Kilduff L, Cook C, Cunningham D, Bunce P, Bracken R (2012). Scaling strength and power for body mass differences in rugby union players. J Sports Med Phys Fitness.

[56] Vaz L, Morais T, Rocha H, James N (2014). Fitness profiles of elite Portuguese rugby union players. J Hum Kinet.

[57] Pumpa KL, Murphy J, Corish CA, Martin W, Ruth E (2012). Anthropometric and body composition analysis: the comparison between different positions and competition levels of successful rugby union players. Int J Body Compos Res.

[58] Vaz L, Vasilica I, Carreras D, Kraak W, Nakamura FY (2016). Physical fitness profiles of elite under-19 rugby union players. J Sports Med Phys Fitness.

[59] Lombard WP, Durandt JJ, Masimla H, Green M, Lambert MI (2015). Changes in body size and physical characteristics of South African under-20 rugby union players over a 13-year period. J Strength Cond Res.

[60] Darrall-Jones J, Roe G, Carney S, Clayton R, Phibbs P, Read D (2016). The effect of body mass on the 30-15 intermittent fitness test in rugby union players. Int J Sports Physiol Perform.

[61] Darrall-Jones JD, Jones B, Roe G, Till K (2016). Reliability and usefulness of linear sprint testing in adolescent rugby union and league players. J Strength Cond Res.

[62] Darrall-Jones JD, Jones B, Till K (2015). Anthropometric and physical profiles of english academy rugby union players. J Strength Cond Res.

[63] Darrall-Jones JD, Jones B, Till K (2016). Anthropometric, sprint, and high-intensity running profiles of english academy rugby union players by position. J Strength Cond Res.

[64] Howard SM, Cumming SP, Atkinson M, Malina RM (2016). Biological maturity-associated variance in peak power output and momentum in academy rugby union players. Eur J Sport Sci.

[65] Jones B, Weaving D, Tee J, Darrall-Jones J, Weakley J, Phibbs P (2018). Bigger, stronger, faster, fitter: the differences in physical qualities of school and academy rugby union players. Journal of Sport Sci.

[66] Weakley Jonathon J.S., Wilson Kyle M., Till Kevin, Read Dale B., Darrall-Jones Joshua, Roe Gregory A.B., Phibbs Padraic J., Jones Ben (2019). Visual Feedback Attenuates Mean Concentric Barbell Velocity Loss and Improves Motivation, Competitiveness, and Perceived Workload in Male Adolescent Athletes. Journal of Strength and Conditioning Research.

[67] Weakley JJS, Till K, Darrall-Jones J, Roe GAB, Phibbs PJ, Read DB (2017). The influence of resistance training experience on the between-day reliability of commonly used strength measures in male youth athletes. J Strength Cond Res.

[68] Till K, Scantlebury S, Jones B (2017). Anthropometric and physical qualities of elite male youth rugby league players. Sports Med.

[69] Sedeaud A, Marc A, Schipman J, Tafflet M, Hager J-P, Toussaint J-F (2012). How they won Rugby World Cup through height, mass and collective experience. Br J Sports Med.

[70] Patton DA, McIntosh AS, Denny G (2016). A review of the anthropometric characteristics, grading and dispensation of junior and youth rugby union players in Australia. Sports Med.

[71] Delahunt E, Byrne RB, Doolin RK, McInerney RG, Ruddock CT, Green BS (2013). Anthropometric profile and body composition of Irish adolescent rugby union players aged 16–18. J Strength and Cond Res.

[72] Smart D, Hopkins WG, Quarrie KL, Gill N (2014). The relationship between physical fitness and game behaviours in rugby union players. Eur J Sport Sci.

[73] Crewther BT, McGuigan MR, Gill ND (2011). The ratio and allometric scaling of speed, power, and strength in elite male rugby union players. J Strength and Cond Red.

[74] Cunningham D, West D, Owen N, Shearer D, Finn C, Bracken R (2013). Strength and power predictors of sprinting performance in professional rugby players. J Sports Med Phys Fitness.

[75] Duthie GM (2006). A framework for the physical development of elite rugby union players. Int J Sports Physiol Perform.

[76] Cobley S, Till K, SC JB, Schorer J, Wattie N (2017). Longitudinal tracking of athlete development: its importance, methods and future considerations. Handbook of Talent Identification and Development in Sport.

[77] Till K, Jones B, Darrall-Jones J, Emmonds S, Cooke C (2015). Longitudinal development of anthropometric and physical characteristics within academy rugby league players. J Strength and Cond Res.

[78] Wehbe G, Gabbett T, Dwyer D, McLellan C, Coad S (2015). Monitoring neuromuscular fatigue in team-sport athletes using a cycle-ergometer test. Int J Sports Physiol Perform.

[79] West DJ, Finn CV, Cunningham DJ, Shearer DA, Jones MR, Harrington BJ (2014). Neuromuscular function, hormonal, and mood responses to a professional rugby union match. J Strength and Cond Res.

[80] Shearer DA, Kilduff LP, Finn C, Jones RM, Bracken RM, Mellalieu SD (2015). Measuring recovery in elite rugby players: the brief assessment of mood, endocrine changes, and power. Res Q Exerc Sport.

[81] Smart D, Gill N, Beaven CM, Cook C, Blazevich A (2008). The relationship between changes in interstitial creatine kinase and game-related impacts in rugby union. Br J Sports Med.

[82] Jones MR, West DJ, Harrington BJ, Cook CJ, Bracken RM, Shearer DA (2014). Match play performance characteristics that predict post-match creatine kinase responses in professional rugby union players. BMC Sports Sci Med Rehabil.

[83] Elloumi M, Maso F, Michaux O, Robert A, Lac G (2003). Behaviour of saliva cortisol [C], testosterone [T] and the T/C ratio during a rugby match and during the post-competition recovery days. Eur J Appl Physiol.

[84] Cunniffe B, Hore AJ, Whitcombe DM, Jones KP, Baker JS, Davies B (2010). Time course of changes in immuneoendocrine markers following an international rugby game. Eur J Appl Physiol.

[85] Oliver JL, Lloyd RS, Whitney A (2015). Monitoring of in-season neuromuscular and perceptual fatigue in youth rugby players. Eur J Sport Sci.

[86] Tee J, Till K, Jones B (2017). Effects of an intensified competition period on neuromuscular function in youth rugby union players. Sport Perf Sci Report.

[87] Roe GA, Phibbs PJ, Till K, Jones BL, Read DB, Weakley JJ (2016). Changes in adductor strength after competition in Academy Rugby Union Players. J Strength and Cond Res.

[88] Roe G, Darrall-Jones J, Till K, Phibbs P, Read D, Weakley J (2017). The effect of physical contact on changes in fatigue markers following rugby union field-based training. Eur J Sport Sci.

[89] Johnston M, Johnston J, Cook CJ, Costley L, Kilgallon M, Kilduff LP (2017). The effect of session order on the physiological, neuromuscular, and endocrine responses to maximal speed and weight training sessions over a 24-h period. J Sci Med Sport.

[90] Noon M, James R, Clarke N, Taylor R, Thake C (2018). Next day subjective and objective recovery indices following acute low and high training loads in Academy Rugby Union Players. Sports..

[91] Johnston RD, Gabbett TJ, Jenkins DG (2015). Influence of playing standard and physical fitness on activity profiles and post-match fatigue during intensified junior rugby league competition. Sports medicine-open.

[92] Desbrow B, McCormack J, Burke LM, Cox GR, Fallon K, Hislop M (2014). Sports Dietitians Australia position statement: sports nutrition for the adolescent athlete. Int J Sport Nutr Exerc Metab.

[93] Black KE, Black AD, Baker DF (2018). Macronutrient intakes of male rugby union players: a review. Int J Sport Nutr Exerc Metab.

[94] Dziedzic CE, Higham DG (2014). Performance nutrition guidelines for international rugby sevens tournaments. Int J Sport Nutr Exerc Metab.

[95] Smith DR, Jones B, Sutton L, King RF, Duckworth LC (2016). Dietary intakes of elite 14-to 19-year-old English academy rugby players during a pre-season training period. Int J Sport Nutr Exerc Metab.

[96] Smith DR, King R, Duckworth L, Sutton L, Preston T, O’Hara J (2018). Energy expenditure of rugby players during a 14-day in-season period, measured using doubly labelled water. Eur J Appl Physiol.

[97] Roza AM, Shizgal HM (1984). The Harris Benedict equation reevaluated: resting energy requirements and the body cell mass. Am J Clin Nutr.

[98] Costello N, Deighton K, Preston T, Matu J, Rowe J, Jones B (2019). Are professional young rugby league players eating enough? Energy intake, expenditure and balance during a pre-season. Eur J Sport Sci.

[99] Burrows T, Harries S, Williams R, Lum C, Callister R (2016). The diet quality of competitive adolescent male rugby union players with energy balance estimated using different physical activity coefficients. Nutrients..

[100] Costello N, Deighton K, Dyson J, Mckenna J, Jones B (2017). Snap-N-Send: A valid and reliable method for assessing the energy intake of elite adolescent athletes. Eur J Sport Sci.

[101] Rees T, Hardy L, Güllich A, Abernethy B, Côté J, Woodman T (2016). The great British medalists project: a review of current knowledge on the development of the world’s best sporting talent. Sports Med.

[102] Hill A, MacNamara Á, Collins D (2015). Psychobehaviorally based features of effective talent development in Rugby Union: a coach’s perspective. Sport Psychol.

[103] Hill AP, Appleton PR (2011). The predictive ability of the frequency of perfectionistic cognitions, self-oriented perfectionism, and socially prescribed perfectionism in relation to symptoms of burnout in youth rugby players. J Sports Sci.

[104] Madigan DJ, Stoeber J, Forsdyke D, Dayson M, Passfield L (2018). Perfectionism predicts injury in junior athletes: preliminary evidence from a prospective study. J Sports Sci.

[105] Nicholls AR, Polman RC (2007). Stressors, coping, and coping effectiveness among players from the England under-18 rugby union team. J of Sport Behavi.

[106] McCarthy N, Collins D, Court D (2016). Start hard, finish better: further evidence for the reversal of the RAE advantage. J Sports Sci.

[107] Williams JM, Andersen MB (1998). Psychosocial antecedents of sport injury: review and critique of the stress and injury model. J Appl Sports Psychol.

[108] McCarthy N, Collins D (2014). Initial identification & selection bias versus the eventual confirmation of talent: evidence for the benefits of a rocky road?. J Sports Sci.

[109] Carter M (2015). The unknown risks of youth rugby. BMJ..

[110] Freitag A, Kirkwood G, Scharer S, Ofori-Asenso R, Pollock AM (2015). Systematic review of rugby injuries in children and adolescents under 21 years. Br J Sports Med.

[111] Pollock AM, Kirkwood G (2016). Removing contact from school rugby will not turn children into couch potatoes. Br J Sports Med.

[112] Van Mechelen W, Hlobil H, Kemper HCG (1992). Incidence, severity, aetiology and prevention of sports injuries. A review of concepts. Sports Med.

[113] Brooks JHM, Fuller CW, Kemp SPT, Reddin DB (2005). Epidemiology of injuries in English professional rugby union: part 1 match injuries. Br J Sports Med.

[114] Brooks JHM, Fuller CW, Kemp SPT, Reddin DB (2005). Epidemiology of injuries in English professional rugby union: Part 2 training injuries. Br J Sports Med.

[115] Brooks JHM, Fuller CW, Kemp SPT, Reddin DB (2005). A prospective study of injuries and training amongst the England 2003 Rugby World Cup squad. Br J Sports Med.

[116] Roberts SP, Trewartha G, England M, Shaddick G, Stokes KA (2013). Epidemiology of time-loss injuries in English community-level rugby union. BMJ Open.

[117] Roberts SP, Trewartha G, England M, Stokes KA (2014). Incidence and nature of medical attendance injuries in English community rugby union. Orthop J Sports Med.

[118] Roberts SP, Trewartha G, England M, Stokes KA (2015). Collapsed scrums and collision tackles: what is the injury risk?. Br J Sports Med.

[119] Palmer-Green DS, Stokes KA, Fuller CW, England M, Kemp SP, Trewartha G (2013). Match injuries in English youth academy and schools rugby union: an epidemiological study. Am J Sports Med.

[120] Haseler CM, Carmont MR, England M (2010). The epidemiology of injuries in English youth community rugby union. Br J Sports Med.

[121] Barden C, Stokes K (2018). Epidemiology of injury in elite English schoolboy rugby union: a 3-year study comparing different competitions. J Athl Train.

[122] Hislop MD, Stokes KA, Williams S, McKay CD, England ME, Kemp SPT (2017). Reducing musculoskeletal injury and concussion risk in schoolboy rugby players with a pre-activity movement control exercise programme: a cluster randomised controlled trial. Br J Sports Med.

[123] Archbold HAP, Rankin AT, Webb M (2017). RISUS study: Rugby Injury Surveillance in Ulster Schools. Br J Sports Med.

[124] Sewry N, Verhagen E, Lambert M (2018). Trends in time-loss injuries during the 2011-2016 South African Rugby Youth Weeks. Scand J Med Sci Sports.

[125] Preatoni E, Cazzola D, Stokes K, England M, Trewartha G (2016). Pre-binding prior to full engagement improves loading conditions for front-row players in contested rugby union scrums. Scand J Med Sci Sports.

[126] Cazzola D, Preatoni E, Stokes KA, England ME, Trewartha G (2015). A modified prebind engagement process reduces biomechanical loading on front row players during scrummaging: a cross-sectional study of 11 elite teams. Br J Sports Med.

[127] Brown JC, Gardner-Lubbe S, Lambert MI, Van Mechelen W, Verhagen E (2015). The BokSmart intervention programme is associated with improvements in injury prevention behaviours of rugby union players: an ecological cross-sectional study. Inj Prev.

[128] Brown JC, Gardner-Lubbe S, Lambert MI, van Mechelen W, Verhagen E (2018). Coach-directed education is associated with injury-prevention behaviour in players: an ecological cross-sectional study. Br J Sports Med.

[129] Brown JC, Verhagen E, Knol D, Van Mechelen W, Lambert MI (2016). The effectiveness of the nationwide BokSmart rugby injury prevention program on catastrophic injury rates. Scand J Med Sci Sports.

[130] Gianotti SM, Quarrie KL, Hume PA (2009). Evaluation of RugbySmart: a rugby union community injury prevention programme. J Sci Med Sport.

[131] Marshall SW, Loomis DP, Waller AE, Chalmers DJ, Bird YN, Quarrie KL (2005). Evaluation of protective equipment for prevention of injuries in rugby union. Int J Epidemiol.

[132] Quarrie KL, Gianotti SM, Chalmers DJ, Hopkins WG (2005). An evaluation of mouthguard requirements and dental injuries in New Zealand rugby union. Br J Sports Med.

[133] Andersson Stig Haugsboe, Bahr Roald, Clarsen Benjamin, Myklebust Grethe (2016). Preventing overuse shoulder injuries among throwing athletes: a cluster-randomised controlled trial in 660 elite handball players. British Journal of Sports Medicine.

[134] Niederbracht Y, Shim AL, Sloniger MA, Paternostro-Bayles M, Short TH (2008). Effects of a shoulder injury prevention strength training program on eccentric external rotator muscle strength and glenohumeral joint imbalance in female overhead activity athletes. J Strength Cond Res.

[135] Collins CL, Fletcher EN, Fields SK, Kluchurosky L, Rohrkemper MK, Comstock RD, Cantu RC (2014). Neck strength: a protective factor reducing risk for concussion in high school sports. J Prim Prev.

[136] Maconi F, Venturelli M, Limonta E, Rampichini S, Bisconti AV, Monti E, Longo S, Esposito F, Ce E (2016). Effects of a 12-week neck muscles training on muscle function and perceived level of muscle soreness in amateur rugby players. Sport Sci Health.

[137] Hendricks S, Matthews B, Roode B, Lambert M (2014). Tackler characteristics associated with tackle performance in rugby union. Eur J Sport Sci.

[138] Lacome M, Piscione J, Hager JP, Carling C (2017). Fluctuations in running and skill-related performance in elite rugby union match-play. Eur J Sport Sci.

[139] den Hollander S, Lambert M, Jones B, Hendricks S (2019). Tackle and ruck technique proficiency within academy and senior club rugby union. Eur J Sport Sci.

[140] England Rugby. Half Game. Available from: https://www.englandrugby.com/my-rugby/players/age-grade-rugby/half-game/. 2018. Accessed 22 June 2019.

